# Integrated Profiling of MicroRNAs and mRNAs: MicroRNAs Located on Xq27.3 Associate with Clear Cell Renal Cell Carcinoma

**DOI:** 10.1371/journal.pone.0015224

**Published:** 2010-12-30

**Authors:** Liang Zhou, Jiahao Chen, Zhizhong Li, Xianxin Li, Xueda Hu, Yi Huang, Xiaokun Zhao, Chaozhao Liang, Yong Wang, Liang Sun, Min Shi, Xiaohong Xu, Feng Shen, Maoshan Chen, Zujing Han, Zhiyu Peng, Qingna Zhai, Jing Chen, Zhongfu Zhang, Ruilin Yang, Jiongxian Ye, Zhichen Guan, Huanming Yang, Yaoting Gui, Jun Wang, Zhiming Cai, Xiuqing Zhang

**Affiliations:** 1 The Key Laboratory of Stem Cell Biology, Guangdong and Shenzhen Key Laboratory of Male Reproductive Medicine and Genetics, Institute of Urology, Peking University Shenzhen Hospital, Shenzhen PKU-HKUST Medical Center, Shenzhen, China; 2 Beijing Genomics Institute at Shenzhen, Shenzhen, China; 3 School of Bioscience and Biotechnology, South China University of Technology, Guangzhou, China; 4 Beijing Institute of Genomics, Chinese Academy of Sciences, Beijing, China; 5 Graduate University of Chinese Academy of Sciences, Beijing, China; 6 Department of Urosurgery, The Second Hospital of Central-Southern University, Changsha, China; 7 Department of Urosurgery, The First Hospital of Anhui Medical University, Hefei, China; 8 Shantou University Medical College, Shantou, China; Baylor College of Medicine, United States of America

## Abstract

**Background:**

With the advent of second-generation sequencing, the expression of gene transcripts can be digitally measured with high accuracy. The purpose of this study was to systematically profile the expression of both mRNA and miRNA genes in clear cell renal cell carcinoma (ccRCC) using massively parallel sequencing technology.

**Methodology:**

The expression of mRNAs and miRNAs were analyzed in tumor tissues and matched normal adjacent tissues obtained from 10 ccRCC patients without distant metastases. In a prevalence screen, some of the most interesting results were validated in a large cohort of ccRCC patients.

**Principal Findings:**

A total of 404 miRNAs and 9,799 mRNAs were detected to be differentially expressed in the 10 ccRCC patients. We also identified 56 novel miRNA candidates in at least two samples. In addition to confirming that canonical cancer genes and miRNAs (including *VEGFA*, *DUSP9* and *ERBB4*; miR-210, miR-184 and miR-206) play pivotal roles in ccRCC development, promising novel candidates (such as *PNCK* and miR-122) without previous annotation in ccRCC carcinogenesis were also discovered in this study. Pathways controlling cell fates (e.g., cell cycle and apoptosis pathways) and cell communication (e.g., focal adhesion and ECM-receptor interaction) were found to be significantly more likely to be disrupted in ccRCC. Additionally, the results of the prevalence screen revealed that the expression of a miRNA gene cluster located on Xq27.3 was consistently downregulated in at least 76.7% of ∼50 ccRCC patients.

**Conclusions:**

Our study provided a two-dimensional map of the mRNA and miRNA expression profiles of ccRCC using deep sequencing technology. Our results indicate that the phenotypic status of ccRCC is characterized by a loss of normal renal function, downregulation of metabolic genes, and upregulation of many signal transduction genes in key pathways. Furthermore, it can be concluded that downregulation of miRNA genes clustered on Xq27.3 is associated with ccRCC.

## Introduction

As the most common type of kidney cancer in adults, renal cell carcinoma (RCC) is responsible for approximately 90% of all cases [1]. RCC is a heterogeneous disease consisting of a number of different subtypes of cancers originating in this organ [2]. Clear cell renal cell carcinoma (ccRCC) accounts for the vast majority (∼70%) of RCC [3]. The five-year survival rates of RCC patients decline considerably as the disease progresses from a localized, regional tumor to distantly metastatic cancer [4].

Despite the high incidence of this type of malignancy in populations of different ethnicities throughout the world, the precise pathogenic mechanisms underlying ccRCC have not been clearly elucidated. Environmental factors, such as cigarette smoking, have been suggested to be associated with increased susceptibility for renal cancer in over 35% of male patients [5], other risk factors include obesity, hypertension, and acquired cystic kidney disease is associated with end-stage renal disease [6]. Molecular studies have identified several genes that are causally implicated in the carcinogenesis of ccRCC, including von Hippel-Lindau (*VHL*) [7], hypoxia-inducible factor-1a (*HIF1a*) [8], vascular endothelial growth factor (*VEGF*), and epidermal growth factor receptor (*EGFR*) [9]. Recently, various genome-wide gene expression profiling studies using microarray-based approaches have provided us with abundant information on the phenotypic characteristics of ccRCC [10], [11], [12]. Nevertheless, few gene expression (profiling) studies have been focused on the mechanisms that drive acquiring the malignancy of ccRCC.

MicroRNA (miRNA) is an important class of small non-coding RNAs that can regulate the expression of protein-coding genes through various mechanisms, including targeted mRNA degradation and translational inhibition [13], [14]. Mutated or abnormally expressed miRNAs have been identified as oncogenes or tumor suppressors in many human cancers [15], including RCC [10], [12], [16]. However, no consistent conclusion could be drawn from most of the previous microarray-based studies due to the limitations of inter-platform differences and the relatively small sample sizes investigated [10], [12], [17], [18]. With the advent of second-generation sequencing technology, the expression level of both miRNAs and mRNAs can be reliably and accurately quantified on whole-genome scale [19]. Additionally, novel miRNAs and mRNAs without previous annotation in public databases can also be discovered with these sequencing platforms [20].

In this report, we present an integrative analysis of digital gene expression (DGE) profiling of both mRNAs and miRNAs in ccRCC by initially detecting their expression levels in 10 matched tumor-normal (adjacent) tissue pairs using the Illumina GA II platform and then validating some of our most interesting findings in a large cohort of ccRCC patients. Furthermore, network analysis of the deregulated genes or the well annotated target genes of the differentially expressed miRNAs pinpointed key signaling and metabolic pathways that were frequently disrupted in most of the investigated ccRCC patients.

## Results

### Overview of the DGE data generated in 10 ccRCCs

**Figure 1:**
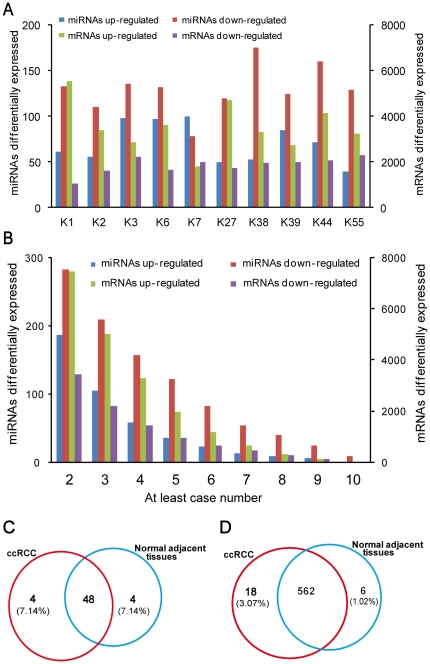
Overview of the expression profiles of miRNAs and mRNAs in 10 ccRCC patients. A: The number of miRNAs and mRNAs differentially expressed in 10 ccRCC patients (*P*<0.01, FDR≤0.001). B: The number of miRNAs and mRNAs recurrently deregulated across 10 ccRCC patients. One miRNA or mRNA may be deregulated only in partial of the 10 patients, and X-axis represents the at least case number, for example, the column 2 represents the number of miRNAs or mRNAs deregulated in at least 2 patients. C: Venn diagram of putative novel miRNA candidates identified in different tissues. D: Venn diagram of putative novel mRNA candidates identified in different tissues.

The expression levels of 17,595 protein-coding genes and 726 human miRNAs were determined to be detectable (at least one transcript per million tags, 1 TPM) in at least one sample (either in tumors or normal adjacent tissues) by our analysis pipeline. A total of 404 distinct miRNAs and 9,799 mRNAs (Figure 1A) were differentially expressed (*P*<0.01, *FDR*≤0.001) in ccRCC samples compared to the matched normal controls (see Table S1 and S2 for details), of which a large proportion was synchronously upregulated or downregulated in at least two patients (Figure 1B). Overall, the percentages of mRNA transcripts upregulated in each individual ranged from 47.5 to 84.1% (average 64.3%), while those for miRNAs ranged from 23.2% to 56.2% (average 35.3%). In addition, 56 potentially novel miRNAs and 586 novel mRNA expression tags were recurrently detected in at least two samples (Tables S3 and S4). There were four novel candidates that were only expressed at detectable levels in the tumor samples, while 40 of the novel miRNAs detectable in both tumor and normal tissues displayed differential expression patterns (FDR ≤0.01) (Figure 1C and Figure 1D).

### Validation of the DGE analysis results using real-time quantitative PCR (qPCR)

**Figure 2:**
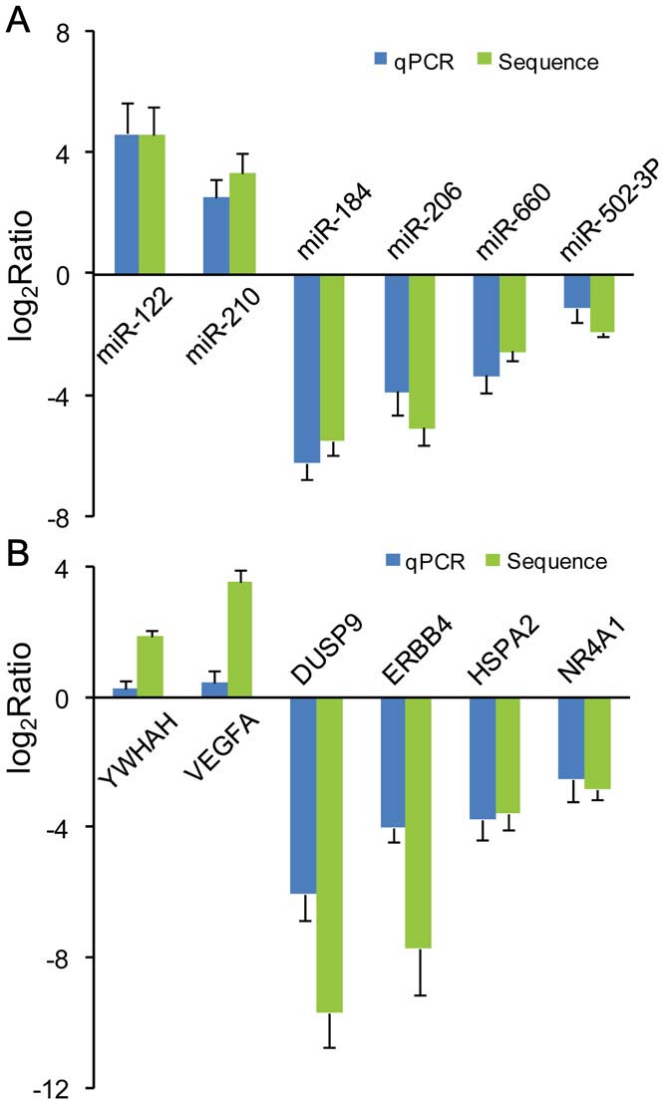
Comparison of deep sequencing data and qPCR results. For the comparison of deep sequencing data and qPCR results, genes determined to be differentially expressed in all of the 10 patients by deep sequencing were validated using qPCR. The height of the columns in the chart represents the log-transformed average fold change (tumor/normal) in expression across the 10 patients for each of the genes validated; bars represent standard errors. A: The validation results of six miRNAs indicated that the deep sequencing data were in excellent agreement with the qPCR results. B: The validation results of six mRNAs also indicated that the results of the deep sequencing were generally agreed well with the qPCR results.

To validate the expression levels of the known miRNA and mRNA genes determined by the DGE analysis, qPCR primers were selectively designed for six miRNA and six mRNA genes (Figure 2A and 2B), each of which was characterized by concordant significant deregulation of its expression across all of the tumor-normal tissue pairs profiled. The resulting validation rates for all of the genes in individual patients ranged from 60% to 100%, with an average success rate of 88.3%. As illustrated in Figure 2A and Figure 2B, the DGE results correlated well with the qPCR results in terms of quantifying the expression of both mRNAs and miRNAs. Additionally, 10 out of 14 of the predicted miRNA candidates identified in at least two samples could be successfully amplified by qPCR using miScript Reverse Transcription and miScript SYBR Green PCR Kits (Qiagen, Germany). Furthermore, the sequences of four out of five randomly selected amplified products (hsa-Np-miR-02, hsa-Np-miR-31, hsa-Np-miR-22, and hsa-Np-miR-15, see Table S3) were confirmed by cloned Sanger sequencing (see Methods for details) as predicted, indicating good accuracy of the DGE profiling in identifying miRNAs (candidates) with no previously described sequence or secondary structure information (Figure S1).

### Deregulated expression of miRNAs and mRNAs in 10 ccRCCs

Consistent with recently published studies, the expression profiles of protein-coding genes in ccRCC were characterized by a loss of expression of most genes that are responsible for normal renal function [21], [22]. For instance, we observed that genes (such as *UMOD* and *AQP2*) or gene families (*SLC22A*) involved in regulating the homeostasis of water-electrolyte balances or transepithelial transportation of toxic organic anions were most significantly downregulated in ccRCCs [23], [24]. In addition, other well-known cancer-associated genes (including *VEGFA*, *DUSP9* and *ERBB4*) were also selectively validated as exhibiting consistently disrupted expression patterns in most of the ccRCC samples profiled (Figure 2B). It should be noted that *PNCK* (average log_2_Ratio  = 8.6) was ranked as the most highly overexpressed gene in all of the tumors in our study.

Among the miRNAs that were most consistently downregulated in the majority of ccRCCs, miR-184 and miR-206 (Figure 2A) were previously reported to promote tumor cell apoptosis via targeting key components in signal transduction pathways [25], [26]. Additionally, consistent with what has been found in other studies [27], [28], miR-210 (a well-established miRNA gene that can be induced under hypoxia conditions in many solid cancers in a *HIF-1a*- and *VHL-*dependent manner) was also found to be significantly overexpressed in all of the ccRCC samples in our study. Paradoxically, though it was the most significantly upregulated miRNA and was validated to be overexpressed nearly 25 fold on average in all 10 of the ccRCCs sequenced, miR-122 has predominately been demonstrated to be significantly downregulated in hepatocellular carcinoma and to act as a negative regulator of tumorigenesis [29]. Thus, full elucidation of the role of miR-122 in ccRCC development beyond its well-known tumor-suppressing function still awaits further study.

As listed in Table S5, a substantial number of miRNAs that were recurrently up- or downregulated in the 10 ccRCCs also displayed deregulated expression patterns in other cancers. The expression of miR-155 has also been found to be upregulated in prediatric Burkitt lymphoma [30], Hodgkin disease [31], CLL [32], AML [33], breast cancer [34] and lung cancer [35]. The possible oncogenic effect of miR-21 has also been reported in other solid cancers (including pancreas, prostate, stomach, colon, lung and breast cancer) [36]. Our study also further confirmed the results presented by Liu *et al.* that the loss of miR-200c expression results in gain of function of *VEGFA*, and increased levels of miR-224 cause the loss of function of the tumor suppressor *ERBB4* in ccRCC [18]. Furthermore, five members of the miR-200 family (including miR-200c, miR-141, miR-200a, miR-200b, and miR-429) were all significantly downregulated in the renal tumors sequenced. Recent studies revealed that the miR-200 family may play a critical role in determining the process of epithelial-to-mesenchymal transition (EMT), and that inhibition of the expression of these miRNAs promoted tumor invasion and migration [37], [38], [39].

### Pathway analysis of differentially expressed genes and miRNA targets in ccRCC

**Figure 3:**
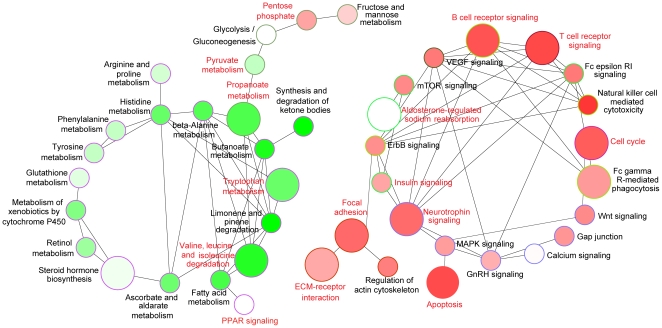
Network analysis of differentially expressed pathways. Nodes in the network represent individual pathways, and edges in the pathway represent the functional relationships between pathways. Pathways significantly enriched with more up- or downregulated genes are represented in red or green, respectively. The color gradient of each node is proportional to the percent of genes up- or downregulated in each pathway. In particular, if there are equal numbers of genes up- and downregulated in a pathway, the node representing the pathway is colored white. The node size reflects the relative degree of significance to which the pathway is enriched in deregulated genes within the interconnected subnetwork. In other words, larger nodes are expected to play more important roles in the interconnected pathway subnetwork. In addition, if a pathway is significantly enriched with differentially expressed genes (corrected *P*-value <0.05), the name of the pathway is highlighted in red (see Table S7 for details).

Interconnected KEGG [40] pathways enriched in deregulated genes were visualized with network-based approaches using Cytoscape [41] with the ClueGo plug-in [42]. As shown in Figure 3, multiple canonical cancer-associated signaling pathways (Table S6), including focal adhesion, cell cycle and ECM-receptor interaction, were significantly more likely to be disrupted in ccRCC than expected by chance (FDR <0.01). Various metabolic pathways (such as those involved in amino acid metabolism) were also observed to be enriched in deregulated genes in our study, (FDR <0.01). Generally, we observed that functionally interacting pathways tended to exhibit similar deregulated expression patterns. As has been found by other investigators, genes regulating cellular signaling were more likely to be overexpressed, whereas metabolic genes were predominantly downregulated in ccRCC [43].

**Figure 4:**
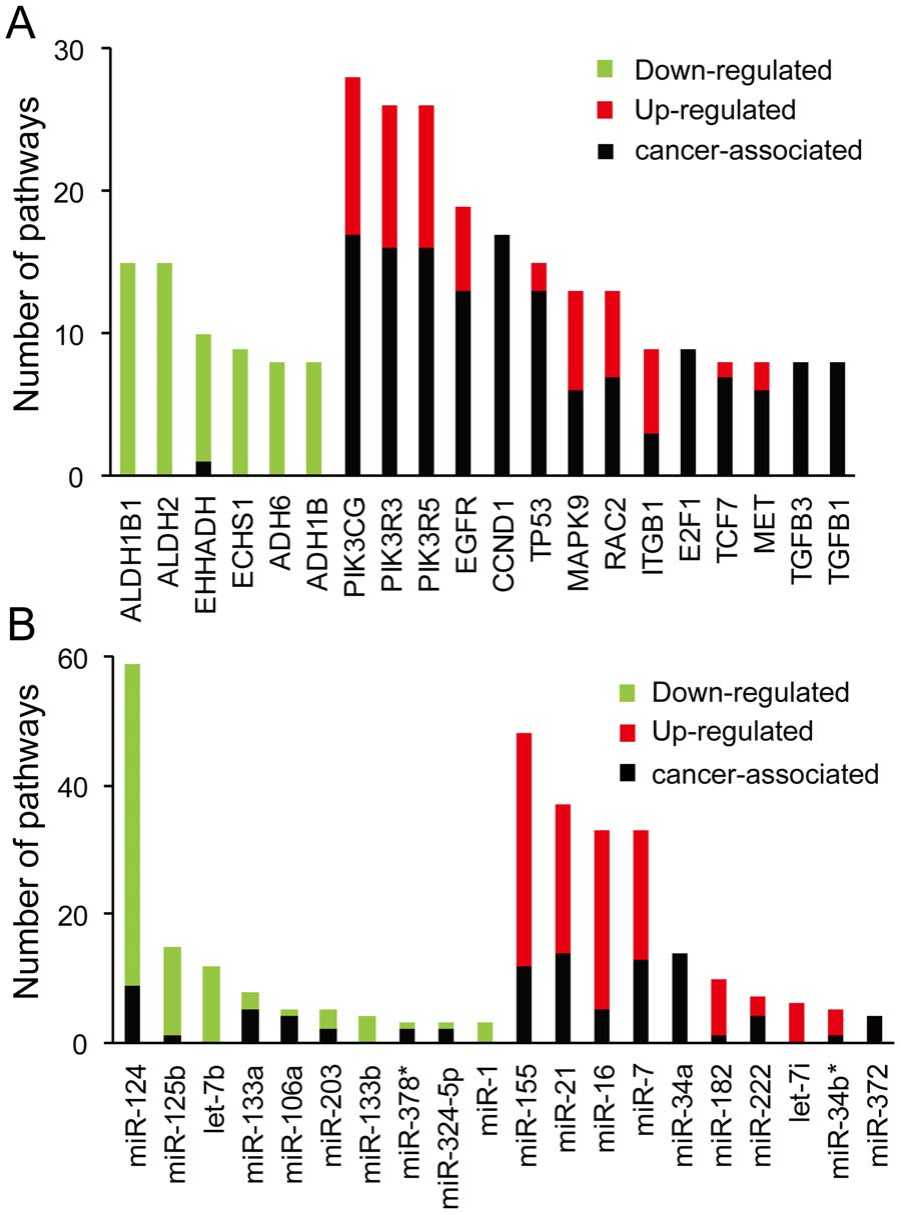
Pathway-based gene set enrichment analyses of differentially expressed mRNAs or miRNAs. Protein-coding genes were ranked according to the number of pathways in which they were prioritized as core genes (most significantly deregulated genes). miRNAs were ranked according to the number of pathways in which their target genes were prioritized as core genes. Columns in green/red represent the genes or miRNAs that were down/upregulated on average in at least two ccRCCs respectively; columns in black represent the genes or miRNA targets involved in at least one cancer-associated pathway. The height of the columns in different colors represents the number of pathways where the genes or miRNA targets were ranked as core genes. A: Genes ranked in the top 20 based on the results of pathway-based gene set enrichment analysis. B: miRNAs ranked the top 20 based on the results of pathway-based gene set enrichment analysis of their target genes.

All KEGG genes that were differentially expressed in at least two patients were prioritized against each pathway by gene set enrichment analysis (GSEA) [44], [45] according to their expression levels in all the samples. Core genes ranked at the top or bottom of each pathway gene set were sorted using the leading edge analysis method introduced by Subramanian A. Figure 4A provides a reduced overview of the common core genes shared by multiple pathways. Previously well-established cancer genes (including *PIK3CG*, *PIK3R3* and *PIK3R5*, *EGFR*, *CCND1* and *TP53*), as well as genes encoding key enzymes (*ALDH1B1*and *ALDH2*) were predicted to play essential roles in ccRCC development based on integrated pathway analysis.

Similarly, target genes of all of the miRNAs deregulated in at least two ccRCCs were retrieved from two experimentally supported databases, TarBase [46] and miRecords [47], for subsequent pathway analysis. Multiple cancer-associated KEGG pathways (e.g., cell cycle and p53 signaling) were found to be enriched in the target genes regulated by miRNAs that were recurrently deregulated in our study (Table S7). Furthermore, all of the targets of recurrently deregulated miRNAs were tested against a smaller pathway set: all 29 pathways catalogued under the hierarchical designation of ‘pathways in cancer’ in the KEGG database using GSEA. As shown in Figure 4B, miR-124 target genes were implicated in the leading edge of 59 pathways, including 9 pathways in cancer, followed by miR-155 (48 pathways, including 12 pathways in cancer) and miR-21 (37 pathways, including 14 pathways in cancer).

### miRNA genes located on Xq27.3 significantly downregulated in ccRCC

**Figure 5:**
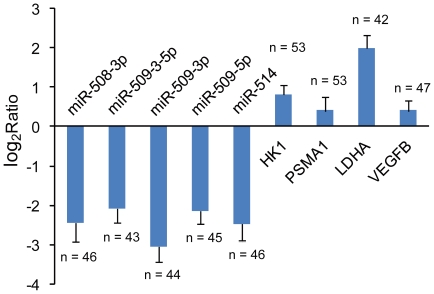
qPCR results for five miRNAs clustered on Xq27.3 and their putative target genes in ∼50 ccRCC patients. The expression of five miRNAs clustered on Xq27.3, as well as the expression of some of their most interesting predicted targets was evaluated in a large sample panel. The height of the columns in the chart represents the log-transformed average fold change (tumor/normal) in expression across all patients for each of the genes validated; bars represent the standard errors. The number of samples (n) used in the validation assay is shown beside each standard error bar. Generally, miRNAs were downregulated in 76.7% to 88.6% of the patients, while the target genes were upregulated in 63.8% to 84.9% of the patients.

Seven miRNAs of the miR-506 family (miR-506, miR508-3p and miR-509-5p, miR-509-3p, miR-509-3-5p, miR-510 and miR-514) were clearly downregulated in most of the renal tumors sequenced in the discovery screen. These miRNA genes were tandemly clustered in the same genomic region, Xq27.3 (∼8 Mb away from the telomere). However, little information on the target genes regulated by these miRNAs could be obtained from current experimentally supported databases (TarBase and miRecords). Nevertheless, as a pilot study, all 23 of the putative targets uniformly predicted by miRanda [48] and miRNAMap [49] were evaluated for their expression in the 10 ccRCCs. On average, all of these targets were clearly upregulated in the tumors in comparison to their matched controls. Predicted targets, such as *VEGFB* and *PSMA1* (regulated by miR-506), *LDHA* (regulated by miR-508-3p) and *HK1* (regulated by miR-509-3p), are core genes involved in multiple key pathways. To further investigate the expression patterns of the miRNA cluster found in the discovery screen, qPCR tests were performed in matched pairs of cancer-normal adjacent tissues in a large panel of ccRCC patients in the prevalence screen. As shown in Figure 5, the expression of all of the tested miRNAs was significantly downregulated in the majority of the tumors screened. In contrast, the expression levels of four predicted targets (*HK1*, *LDHA*, *PSMA1*and *VEGFB*) were upregulated on average.

## Discussion

DGE sequencing is a powerful and reliable tool for accurately quantifying the absolute expression levels of both mRNAs and miRNAs and overcomes the drawbacks of detecting a limited number of relatively high-abundance transcripts determined by the availability of array probes [20]. The results of deep sequencing correlated well with those of qPCR in our study based on the validation results of randomly selected miRNA and mRNA genes. In addition, the novel miRNA candidates identified in this study, especially those validated by qPCR and Sanger sequencing, further complements the current human miRNA catalog.

The most significantly downregulated gene in ccRCC in comparison to normal tissue was the pregnancy-up-regulated non-ubiquitously expressed CaM kinase gene (*PNCK*). *PNCK* was previously found to be overexpressed in a particular subtype of epithelial cells involved in the differentiation and transformation of breast cancer [50]. A recent report also revealed that PNCK mediates the protea-lysosomal degradation of EGFR protein and may represent a promising target for therapeutic intervention in *EGFR*-regulated oncogenesis [51]. Here, we report its possible association with the development or progression of ccRCC for the first time, though the molecular mechanisms underlying this association remain largely unknown. Other well-known oncogenes that control the cell division cycle, such as *CDCA2* and *CDKN2A*, were also likely to play crucial roles in the development of ccRCC, as they were ranked at the top of the lists of genes that were highly overexpressed in most ccRCCs in this study.

Our study highlights the importance of investigating the functional roles of miRNAs in the tumorigenesis of ccRCC. Of the minority (35.3%) of miRNAs that were most significantly upregulated in the tumor tissues, the outlier miR-122 was initially identified as a tumor-suppressor in other cancers [52], [53], indicating the necessity for the intensive investigation of well-known cancer-associated miRNAs in different kinds of tumors for full elucidation of their functional roles in tumorigenesis. Cellular adaptation to hypoxic microenvironments is a characteristic of many solid cancers [54]. Genetic inactivation of the *VHL* gene, which regulates the ubiquitin-ligated degradation of hypoxia-inducible factors (HIFs), has frequently been reported as the “driving event” initiating ccRCC [55]. Accumulation of *HIF-1a* at the protein level leads to continuous activation of downstream target genes (e.g., *VEGF* and *PDGF*), which were all upregulated in our tumor sample panel [56], [57]. Interestingly, miR-21, one of the few currently known miRNA genes under the control of the *VHL*-*HIF-1a* cascade, was also found to be significantly overexpressed in ccRCC. It can be speculated that characterization of the miRNA genes targeted by the *VHL*-HIF cascade, or the protein-coding genes targeted by the *VHL*-HIF-miRNA cascade would be of great value in furthering our understanding of the pathogenic mechanisms underlying ccRCC.

It has become widely acknowledged that disrupted pathways, as opposed to deregulated individual gene, drive the tumorigenesis process [58]. Network-based pathway analysis of significantly deregulated protein-coding genes allowed us to obtain an overview of key pathways that may have profound effects on ccRCC development. Core genes common to multiple pathways are more likely to be cancer genes. As shown in Figure 4A, most of the genes ranked in the top 20 of the final core gene sets determined by GSEA have previously been causatively linked with tumorigenesis in other studies, indicating a high predictive value of this integrative pathway analysis in identifying genuine cancer genes in ccRCC using our sequencing data. Similarly, all significantly deregulated miRNA genes were also prioritized based on integrative pathway analysis of their target genes retrieved from experimentally supported databases. miR-155, miR-21, miR-34a, miR-135a and miR-135b were previously found to be associated with ccRCC [12], [18], [59], indicating that they are authentic cancer-associated miRNAs in ccRCC. miR-155 and miR-21 were also reported to be involved in the development of different cancers [30], [31], [32], [33], [34], [35], [36]. In summary, in addition to the miRNA and mRNA genes known to implicate in cancer development, other genes prioritized at the top of our core gene list are promising candidates worthy of further investigation.

It has been proposed that most of the known miRNAs are tandemly clustered [60], [61] and are transcribed as polycistronic primary transcripts [62]. Positional enrichment analysis of the deregulated miRNA genes revealed that the expression levels of the miRNAs clustered at the fragile site Xq27.3 [63] were significantly decreased in our study samples. Interestingly, careful analysis of the results of another small sample study on ccRCC also revealed that five of the seven miRNAs were downregulated in tumor tissues [64]. However, little attention has been paid to identifying the target genes regulated by these miRNAs using classic molecular biology methods. In the prevalence screen conducted in this study, the deregulated expression patterns detected in ∼50 ccRCC patients for five of the miRNAs further supported the finding that the miRNA genes located on Xq27.3 were expressed at substantially lower levels in at least 76.7% of primary ccRCCs compared to patient-matched normal adjacent controls. The high prevalence of this abnormal expression pattern suggested that the loss of expression of the miRNAs clustered at Xq27.3 is an important event in ccRCC pathogenesis, and more detailed studies are needed to establish their exact functional roles in carcinogenesis.

In summary, this is one of the few studies that have simultaneously profiled the expression patterns of both miRNAs and mRNAs on a genome-wide scale in ccRCC patients using second-generation sequencing technology. Our results demonstrated that the expression phenotype of ccRCC is characterized by a loss of normal renal function, downregulated expression of metabolic genes, and upregulation of many signal transduction genes in key pathways. Individual pathway enrichment analysis revealed that well-known cancer pathways (e.g., cell cycle, apoptosis, focal adhesion and ECM-receptor interaction) play critical roles in ccRCC development. With the aid of currently available databases, we found that cancer-associated mRNA and miRNA genes could be accurately prioritized with the integrative pathway analysis approach using our sequencing data. Finally, we also established the association of a cluster of miRNA genes on Xq27.3 with ccRCC in a large sample set.

## Materials and Methods

### Clinical sample collection

All of the ccRCC tissues and matched normal adjacent tissues used in this study were obtained from the clinical institutions of Urinogenital Cancer Genomics Consortium (UGCC) in China. Detailed information on the 10 patients sequenced in the discovery screen is summarized in Table S8. Specimens were snap-frozen in liquid nitrogen or deposited in RNALater (Qiagen, Germany) and subsequently stored at −80°C. Hematoxylin-eosin (HE)-stained sections were examined for tumor cell percentage, and tumor tissues containing more than 80% tumor cells were selected for further study. The matched normal adjacent tissues were defined as kidney tissues located 2.0 cm outside of visible ccRCC lesions. Histopathologic examination of the normal tissues indicated the presence of normal renal tubules and glomeruli without tumor cell contamination (Figure S2). The collection and use of the patient samples were reviewed and approved by Institutional Ethics Committees, and written informed consent from all patients was appropriately obtained.

### RNA extraction

Total RNA was extracted from ccRCC and normal adjacent tissues using TRIZOL (Invitrogen, US) according to the manufacture's protocol and evaluated using Agilent 2100 Bioanalyzer (Agilent Technologies, US).

### Digital gene expression (DGE) sequencing of mRNA and statistical analysis

Of the total RNA isolated from each sample, 4 µg was used in DGE sequencing. Briefly, following synthesis of double-stranded cDNA using oligo(dT)_18_ beads, the cDNA was digested with NlaIII and ligated to a first adapter (GEX adapter 1) containing a restriction site recognized by MmeI. After dephosphorylation with alkaline phosphatase CIAP, the purified MmeI-digested products were linked to a second adapter (GEX index adapter) containing 2-bp degenerate 3′ overhangs. Then, the double adapter-flanked tags from the mRNAs were amplified by PCR using Phusion DNA polymerase and Gex PCR primers following the manufacturer's protocol. PCR was carried out using the following program: 98°C for 30 sec, followed by 15 cycles of 98°C for 10 sec, 60°C for 30 sec and 72°C for 15 sec, and then 72°C for 10 min. The resulting ∼85-bp PCR products were ethanol precipitated and purified from electrophoresis gels using Spin-X filter columns. Finally, mRNA libraries were sequenced on the Illumina Cluster Station and Genome Analyzer II (Illumina Inc, USA) following the manufacturer's protocol.

Before statistical analysis, potentially erroneous tags (single copy tags and tags consisting of adapter sequences or containing unknown sequences ‘N’) were filtered out. All of the 17-bp sequences next to the possible Nla III restriction sites in a human reference genome (hg19) and the 4-bp CATG restriction enzyme-digested site were extracted and concatenated as a new reference [65]. Tags were mapped to the constructed reference using SOAP V2.0 [66] allowing no more than one base mismatch. Only unique mapping tags were used for gene expression analysis. Standardized TPM (transcripts per million clean tags) values were applied to compare gene expression between tumors and normal adjacent tissues. The expression fold change (tumor versus normal) for each gene was calculated as the log_2_Ratio using TPM values. Subsequently, we performed a rigorous significance test to determine the differentially expressed genes [67]. The resulting *P*-values for all genes were corrected for multiple tests using a FDR (false discovery rate) adjustment [68]. In addition, after filtering out all annotated mRNA tags and those tags matching the mitochondrial genome, the remaining clean tags that could be mapped to the human reference genome were identified as potentially novel mRNA expression tags. Only those tags expressed in at least two samples at detectable levels (≥1 TPM) were defined as high-confidence novel mRNA expression tags.

### DGE miRNA sequencing and statistical analysis

For miRNA sequencing, 5 µg of total RNA form each sample was ligated with both a 5′ adapter and 3′ adapter for reverse transcription using Superscript II at 42°C for 1 h and 70°C for 15 min. Subsequently, the reverse transcribed products were amplified using the following PCR program: a 15-cycle reaction at 98°C for 30 sec, followed by 15 cycles of 98°C for 10 sec, 72°C for 15 sec, and then 72°C for 10 min. After obtaining a ∼92-bp DNA band on 6% PAGE gels, the PCR products were ethanol precipitated and purified using Spin-X filter columns. Finally, miRNA libraries were sequenced on the Illumina Cluster Station and Genome Analyzer II following the manufacturer's protocol.

Low quality reads were trimmed and adapter sequences were accurately clipped with the aid of a dynamic programming algorithm before subsequent statistical analysis. After elimination of the duplicate reads, the remaining reads of at least 18 nt were mapped to a human reference genome (hg19) using SOAP V2.0. To remove tags originating from protein-coding genes, repeat sequences, rRNA, tRNA, snRNA, and snoRNA, we also mapped the short read tags to UCSC RefGene, RepeatMasker and NCBI Refseq, as well as our in-house ncRNA annotation datasets compiled from the NCBI GenBank database (http://www.ncbi.nih.gov). The same pipeline used for DGE mRNA differential expression analysis was also used for miRNA expression analysis.

### Prediction of novel miRNA candidates and determination of miRNA target genes

Mireap (http://sourceforge.net/projects/mireap) was used to predict novel miRNA candidates based on their secondary structure, stability of their hairpin structure and the Dicer cleavage site of the miRNA tags. Moreover only those candidates fulfilling the following two criteria were defined as high-confidence miRNA candidates in our study: 1) stable hairpin structure with low free energy (<−20 kcal/mol); 2) expressed in at least two samples at detectable levels (1 TPM).

miRNA target genes that were supported by two databases (TarBase and miRecords) with experimental evidence were retrieved for pathway analysis. For those miRNAs (clustered on Xq27.3) without target recorded in the two databases mentioned above, we only retained those putative targets uniformly predicted by miRanda and miRmap 2.0.

### Pathway analysis of differentially expressed genes and miRNAs

KEGG pathway analysis was performed the using Cytoscape V2.6.3 (http://cytoscape.org/) with the ClueGo plug-in. Right-side hypergeometric tests were used to identify pathways enriched in deregulated genes. Benjamini-Hochberg adjustment was applied for multiple test correction. Differentially expressed genes, as well as target genes of deregulated miRNAs were mapped onto KEGG pathways for network construction.

All KEGG genes that were differentially expressed in at least two patients were prioritized against each pathway by gene set enrichment analysis (GSEA) according to their expression levels in all of the samples. Core genes or miRNA targets ranked at the top or bottom of each pathway gene list were most significantly altered between tumors and normal adjacent tissues (called leading edge in GSEA). An individual gene or miRNA target may be implicated in the leading edge of multiple pathways, and the numbers of pathways in which the core genes or miRNAs were implicated were determined by the leading edge analysis. In addition, to further reveal the roles of miRNAs and genes in ccRCC, we subsequently constructed a dataset based on all 29 pathways catalogued under the hierarchical designation of ‘pathways in cancer’ (ko05200) in the KEGG database for GSEA. miRNAs and genes ranked in the leading edge of these cancer-associated pathways may play more important roles than others in tumorigenesis.

### Validation of the expression of miRNAs and mRNAs by qPCR

To validate the expression levels of the known miRNA and mRNA genes determined by deep sequencing, qPCR primers were selectively designed for six miRNA and six mRNA genes. Six genes (*VEGFA*, *YWHAH*, *DUSP9*, *NR4A1*, *HSPA2* and *ERBB4*) that were differentially expressed in all patients in the discovery screen were validated in both tumor and normal adjacent tissues from the 10 ccRCC patients using qPCR. β-actin was selected as the internal control. In brief, 2 µg of total RNA from each sample was reverse transcribed for cDNA synthesis using a reverse transcription kit according to the manufacturer's protocol (Promega, Madison, WI). The reverse transcription products were amplified using the following PCR program: 94°C for 4 min, followed by 30 cycles of 94°C for 30 sec, 55°C for 30 sec, 72°C for 30 sec and then extension at 72°C for 10 min. Six miRNAs (miR-122, miR-210, miR-184, miR-206, miR-660 and miR-502-3p) that were deregulated in all patients were also selected for validation by qPCR using the miScript Reverse Transcription and miScript SYBR Green PCR Kits according to the manufacturer's protocol (Qiagen, Germany) and snRNA U6 was used as the internal control. PCR was performed for both tumor and normal tissue samples using the following program: 95°C for 15 minutes, followed by 40 cycles of 94°C for 15 sec, 55°C for 30 sec and 72°C for 30 sec.

The expression levels of miR-509-5p, miR508-3p, miR-514, miR-509-3-5p and miR-509-3p, as well as their putative target genes (*PSMA1*, *LDHA*, *HK1* and *VEGFB*) were evaluated in both the tumor and normal adjacent tissues obtained from ∼50 ccRCC patients by qPCR. The PCR programs and reverse transcription kits used were as mentioned above.

We randomly selected 14 novel miRNAs candidates for validation by qPCR. Briefly, after adding poly(A) to the end of mature miRNAs with poly(A) polymerase, reverse-transcription was performed using oligo-dT and random primers. Using the resulting cDNA products as templates, real-time PCR was performed with miRNA-specific primers and universal primers provided by the miScript SYBR Green PCR Kit. The purified qPCR products excised from agarose gels were cloned into the PMD18-T vector (TAKARA, Dalian, China) for subsequent sequencing on an AB 3730 DNA analyzer. All of the primers used in the validation assays are listed in Table S9.

## Supporting Information

Figure S1 Validation results of five novel miRNA candidates.
(DOC)

Figure S2 HE staining of tumor and normal adjacent tissues from patient K1.
(DOC)

Table S1 List of differentially expressed miRNAs.
(XLS)

Table S2 List of differentially expressed mRNAs.
(XLS)

Table S3 Detailed information on predicted novel miRNA candidates.
(XLS)

Table S4 Detailed information on unmapped mRNA tags.
(XLS)

Table S5 Comparison of the most significantly deregulated miRNAs with those reported in published studies.
(DOC)

Table S6 Pathway enrichment analysis of differentially expressed genes in ccRCC.
(XLS)

Table S7 Pathway enrichment analysis of genes targeted by differentially expressed miRNAs in ccRCC.
(XLS)

Table S8 Clinical information on the 10 patients sequenced in the discovery screen.
(DOC)

Table S9 qPCR primers used in the validation assays.
(DOC)
