# Supplementary material for: Integrated Profiling of MicroRNAs and mRNAs: MicroRNAs Located on Xq27.3 Associate with Clear Cell Renal Cell Carcinoma
Source: PLoS One. 2010 Dec 30;5(12):e15224. doi: 10.1371/journal.pone.0015224 (PMC3013074; doi:10.1371/journal.pone.0015224)
Supplement: Figure S1 — Validation results of five novel miRNA candidates. (DOC) [file pone.0015224.s001.doc]

**
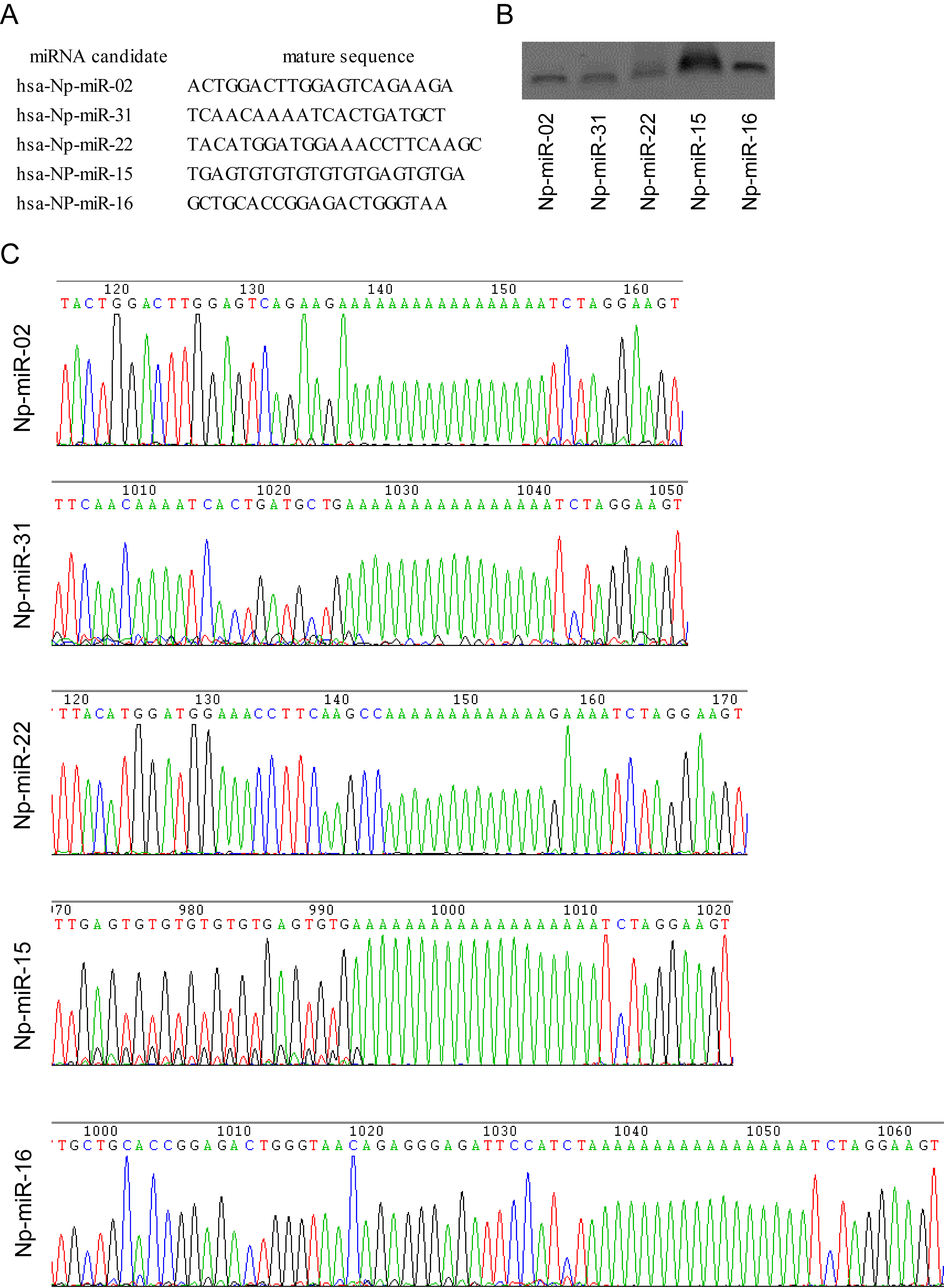
**

**Figure S1. Validation results of five** **novel miRNA candidates.** A: The mature sequences of five novel miRNA candidates. B: qPCR products detected by agarose gel electrophoresis. C: Representative results of cloned Sanger Sequencing for each miRNA candiates.
