# Supplementary material for: Integrated Profiling of MicroRNAs and mRNAs: MicroRNAs Located on Xq27.3 Associate with Clear Cell Renal Cell Carcinoma
Source: PLoS One. 2010 Dec 30;5(12):e15224. doi: 10.1371/journal.pone.0015224 (PMC3013074; doi:10.1371/journal.pone.0015224)
Supplement: Figure S2 — HE staining of tumor and normal adjacent tissues from patient K1. (DOC) [file pone.0015224.s002.doc]

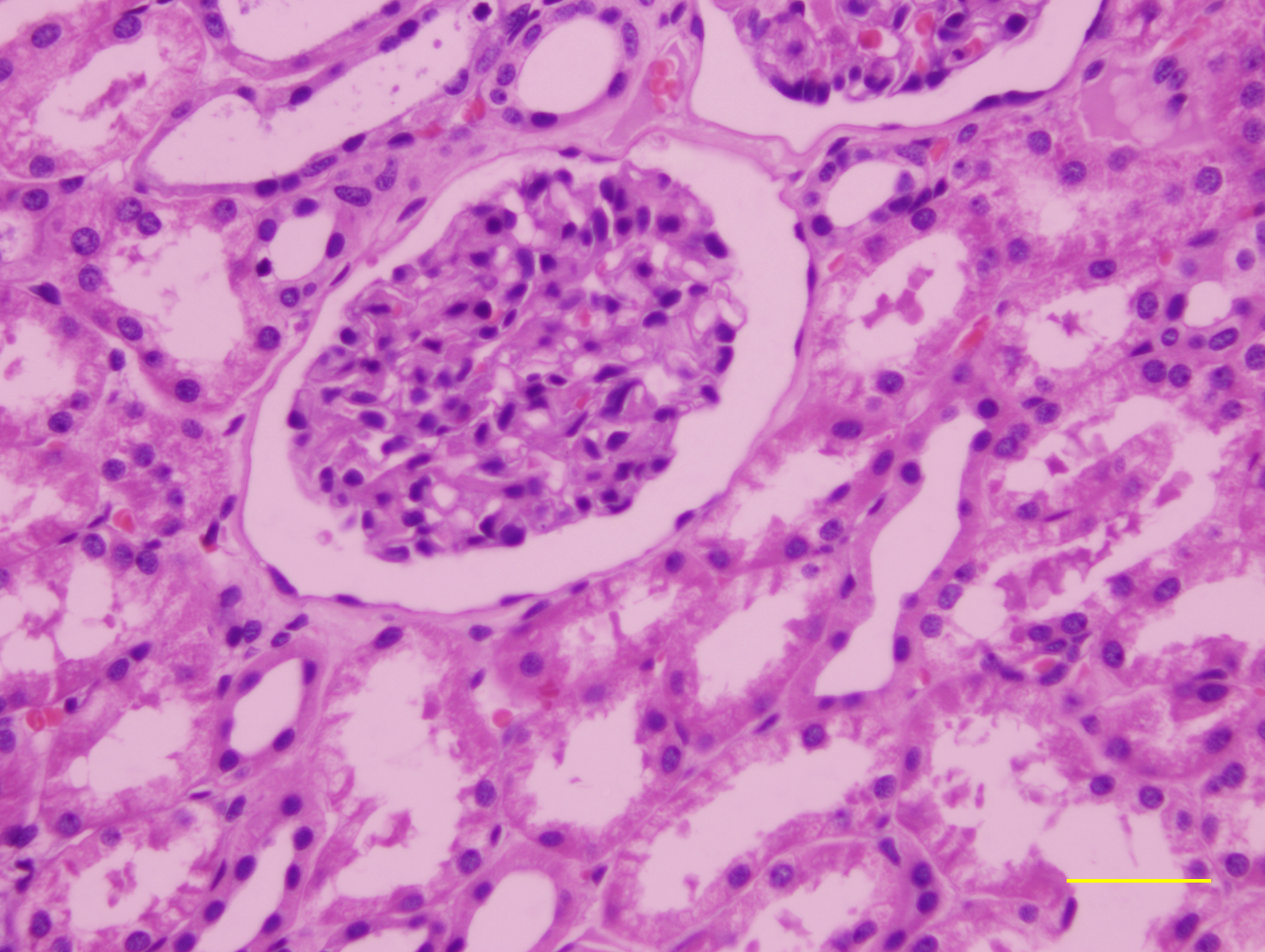

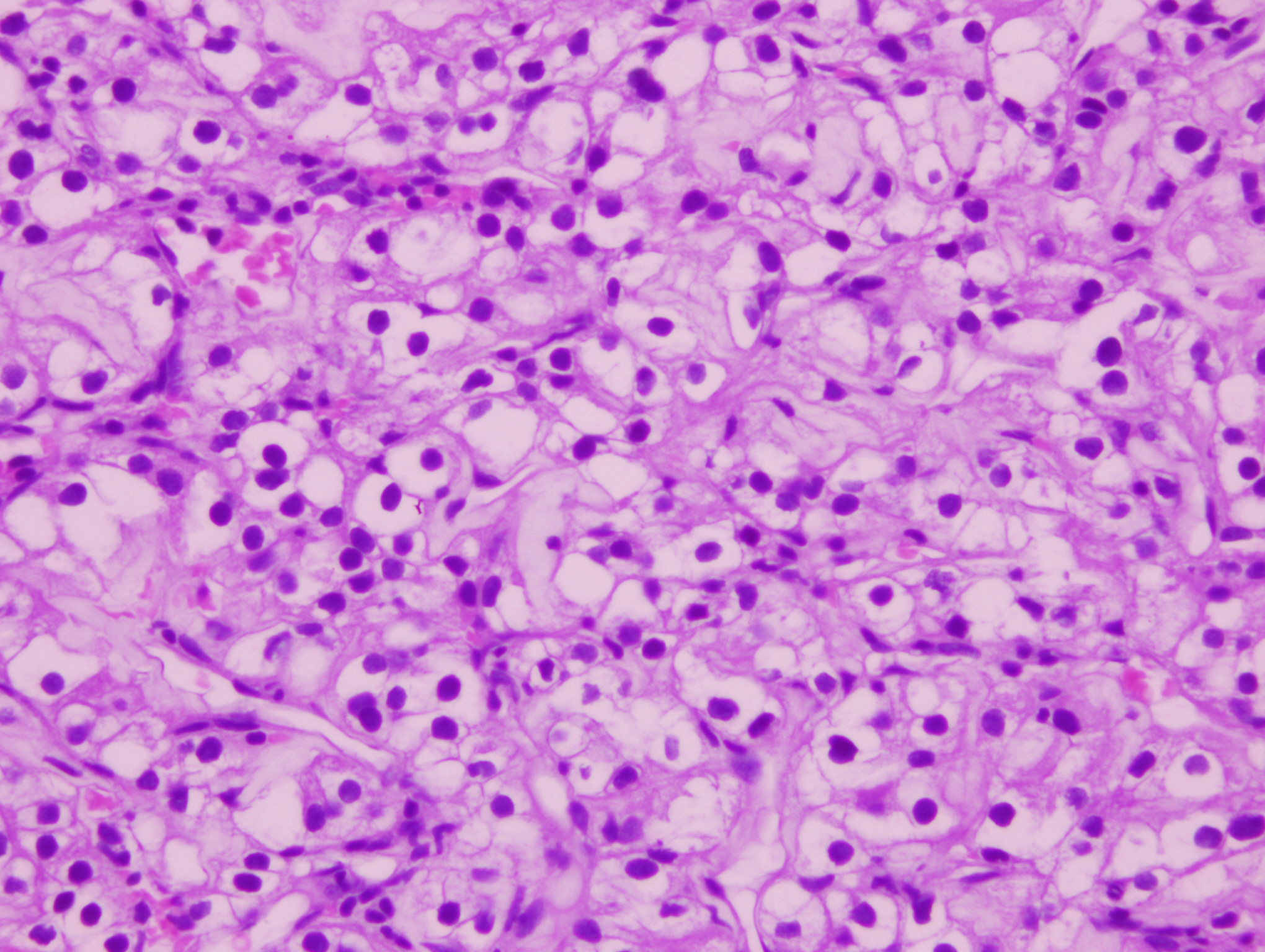
**A**  **B**

**Figure S2.** **HE staining of tumor and normal adjacent tissues from patient K1.**

A: ccRCC tissue. B: normal adjacent tissue.
