# Supplementary material for: Integrated Profiling of MicroRNAs and mRNAs: MicroRNAs Located on Xq27.3 Associate with Clear Cell Renal Cell Carcinoma
Source: PLoS One. 2010 Dec 30;5(12):e15224. doi: 10.1371/journal.pone.0015224 (PMC3013074; doi:10.1371/journal.pone.0015224)
Supplement: Table S5 — Comparison of the most significantly deregulated miRNAs with those reported in published studies. (DOC) [file pone.0015224.s007.doc]

| **Table S5.** Comparison of the most significantly deregulated miRNAs with those reported in published studies. | | | | | | | | | |
| --- | --- | --- | --- | --- | --- | --- | --- | --- | --- |
| **Up-regulated** | | | |  | | **Down-regulated** | | | |
| **miRNA** | **log_2_Ratio^a^** | **Chromosomal location** | **References^b^** | |  | **miRNA** | **log_2_Ratio^a^** | **Chromosomal location** | **References^b^** |
| **122** | 4.6 | 18q21.31 | [5] | |  | **184** | -5.5 | 15q25.1 | [5] |
| **210** | 3.3 | 11p.15.5 | [1,2,3,4,5] | |  | **206** | -5.1 | 6p12.2 | [5] |
| **224** | 2.7 | Xq28 | [1,2,3,4] | |  | **216b** | -5 | 2p16.1 | - |
| **155** | 2.3 | 21q21.3 | [1,2,3,4,5] | |  | **509-5p** | -4.9 | Xq27.3 | [5] |
| **21** | 2.2 | 17q23.1 | [2,3,4] | |  | **508-3p** | -4.9 | Xq27.3 | [5] |
| **144** | 2 | 17q11.2 | - | |  | **514** | -4.7 | Xq27.3 | [1,2,3,4,5] |
| **34a** | 2 | 1p36.22 | [2,3,4] | |  | **509-3-5p** | -4.6 | Xq27.3 | - |
| **886-3p** | 1.8 | 5q31.3 | - | |  | **506** | -4.3 | Xq27.3 | [5] |
| **590-3p** | 1.8 | 7q11.23 | - | |  | **200c** | -4.3 | 12p13.31 | [1,2,3,4,5] |
| **7-1/2/3** | 1.7 | 9q21.32/15q26.1/19p13.3 | - | |  | **204** | -3.7 | 9q21.12 | [2,3] |
| **142-3p** | 1.7 | 17q22 | [2] | |  | **135a-1/2** | -3.7 | 3p21.1/12q23.1 | [2,3] |
| **1308** | 1.6 | Xp22.2-p22.1 | - | |  | **509-3p** | -3.6 | Xq27.3 | [5] |
| **629** | 1.6 | 15q23 | - | |  | **217** | -3.4 | 2p16.1 | - |
| **452** | 1.6 | Xq28 | - | |  | **9-1/2/3** | -3.4 | 1q22/5q14.3/15q26.1 | [5] |
| **885-3p** | 1.4 | 3q25.3 | - | |  | **187** | -3.3 | 18q12.2 | - |
| **142-5p** | 1.3 | 17q22 | [2,3] | |  | **141** | -3.3 | 12p13.31 | [1,2,3,4,5] |
| **592** | 1.3 | 7q31.33 | [2,3,5] | |  | **532-3p** | -3.1 | Xp11.23 | - |
| **16-2*** | 1.3 | 3q25.33 |  | |  | **363** | -3 | Xq26.2 | - |
| **584** | 1.3 | 5q32 | - | |  | **138-1/2** | -2.9 | 3p21.32/16q13 | - |
| **1277** | 1.3 | Xq24 | - | |  | **20b** | -2.8 | Xq26.2 | - |
| ^a^ccRCC versus normal adjacent tissues, FDR≤0.1%, P<0.01. | | | | | | | | | |
| ^b^The expression pattern of these miRNAs in ccRCC were consistent with that reported in the references.  **References** | | | | | | | | | |

1. Jung M, Mollenkopf HJ, Grimm C, Wagner I, Albrecht M, et al. (2009) MicroRNA profiling of clear cell renal cell cancer identifies a robust signature to define renal malignancy. J Cell Mol Med 13: 3918-3928.

2. Liu H, Brannon AR, Reddy AK, Alexe G, Seiler MW, et al. (2010) Identifying mRNA targets of microRNA dysregulated in cancer: with application to clear cell Renal Cell Carcinoma. BMC Syst Biol 4: 51.

3. Juan D, Alexe G, Antes T, Liu H, Madabhushi A, et al. (2009) Identification of a microRNA panel for clear-cell kidney cancer. Urology 75: 835-841.

4. Nakada C, Matsuura K, Tsukamoto Y, Tanigawa M, Yoshimoto T, et al. (2008) Genome-wide microRNA expression profiling in renal cell carcinoma: significant down-regulation of miR-141 and miR-200c. J Pathol 216: 418-427.

5. Weng L, Wu X, Gao H, Mu B, Li X, et al. (2010) MicroRNA profiling of clear cell renal cell carcinoma by whole-genome small RNA deep sequencing of paired frozen and formalin-fixed, paraffin-embedded tissue specimens. J Pathol 222: 41-51.
