# Supplementary material for: Integrated Profiling of MicroRNAs and mRNAs: MicroRNAs Located on Xq27.3 Associate with Clear Cell Renal Cell Carcinoma
Source: PLoS One. 2010 Dec 30;5(12):e15224. doi: 10.1371/journal.pone.0015224 (PMC3013074; doi:10.1371/journal.pone.0015224)
Supplement: Table S8 — Clinical information on the 10 patients sequenced in the discovery screen. (DOC) [file pone.0015224.s010.doc]

**Table S8.** Clinical information on the 10 patients sequenced in the discovery screen

| Patient | Age (years) | Gender | TNM |
| --- | --- | --- | --- |
| K1 | 32 | M | T1N0M0 |
| K2 | 36 | M | T2N0M0 |
| K3 | 41 | M | T2N0M0 |
| K6 | 40 | M | T1N0M0 |
| K7 | 27 | M | T1N0M0 |
| K27 | 52 | F | T2N0M0 |
| K38 | 40 | F | T1N0M0 |
| K39 | 58 | M | T2N0M0 |
| K44 | 48 | F | T2N0M0 |
| K55 | 52 | M | T4N0M0 |
