# Supplementary material for: Integrated Profiling of MicroRNAs and mRNAs: MicroRNAs Located on Xq27.3 Associate with Clear Cell Renal Cell Carcinoma
Source: PLoS One. 2010 Dec 30;5(12):e15224. doi: 10.1371/journal.pone.0015224 (PMC3013074; doi:10.1371/journal.pone.0015224)
Supplement: Table S9 — qPCR primers used in the validation assays. (DOC) [file pone.0015224.s011.doc]

**Table S9.** qPCR primers used in the validation assays

| **Gene/miRNA** | **Primers(5'-3')** |
| --- | --- |
| VEGFA Forward | CGGAAGATTAGAGAGTTT |
| VEGFA Reverse primer | TGAAGACACCAATAACAT |
| YWHAH Forward | GATTACTACCGCTACTTAG |
| YWHAH Reverse primer | GCATTCTGGATCTCATAG |
| DUSP9 Forward | CCATTGAGTTCATTGATG |
| DUSP9 Reverse primer | GATGTTAGACTTCTTCCT |
| NR4A1 Forward | GTAAATACAGGAAGAAAGAG |
| NR4A1 Reverse primer | ATACACAAATATAAATGTCAGA |
| HSPA2 Forward | CTGGAGTCCTATACCTAC |
| HSPA2 Reverse primer | TTCTGCTTGTGTTCATAC |
| ERBB4 Forward | GGAATACTGTGGTGTAAG |
| ERBB4 Reverse primer | TGTTCAAGTTAGGTAAGC |
| hsa-miR-502-3p Forward | ATCCTTGCTATCTGGGTGCTA |
| hsa-miR-660 Forward | TACCCATTGCATATCGGAGTTG |
| hsa- miR-206 Forward | TGGAATGTAAGGAAGTGTGTGG |
| hsa-miR122 Forward | TGGAGTGTGACGGTGGTGTTTG |
| hsa-miR-509-5p Forward | tactgcagacagtggcaatca |
| hsa-miR-508-3p Forward | tgattgtagccttttggagtaga |
| hsa-miR-514 Forward | attgacacttctgtgagtaga |
| hsa-miR-509-3-5p Forward | tactgcagacgtggcaatcatg |
| hsa-miR-506 Forward | taaggcacccttctgagtaga |
| hsa-miR-509-3p Forward | tgattggtacgtctgtgggtag |
| HK1 Forward | aggaaggagatgaagaatg |
| HK1 Reverse primer | aatggaccttacgaatft |
| LDHA Forward | Caaactcaaaggctacacat |
| LDHA Reverse primer | aaggaacactaaggaagaca |
| VEGFB Forward | ggagatgtccctggaagaacac |
| VEGFB Reverse primer | ctgtctggcttcacagcactg |
| PSMA1 Forward | tactgctgatgctagact |
| PSMA1 Reverse primer | tagagatacaagacgagaca |
| hsa-Np-miR-02 | ACTGGACTTGGAGTCAGAAGA |
| hsa-Np-miR-31 | TCAACAAAATCACTGATGCT |
| hsa-Np-miR-22 | TACATGGATGGAAACCTTCAAGC |
| hsa-NP-miR-15 | TGAGTGTGTGTGTGTGAGTGTGA |
| hsa-NP-miR-16 | GCTGCACCGGAGACTGGGTAA |
